# Galectin-1, -3 and -9 Expression and Clinical Significance in Squamous Cervical Cancer

**DOI:** 10.1371/journal.pone.0129119

**Published:** 2015-06-12

**Authors:** Simone Punt, Victor L. Thijssen, Johannes Vrolijk, Cornelis D. de Kroon, Arko Gorter, Ekaterina S. Jordanova

**Affiliations:** 1 Department of Pathology, Leiden University Medical Center, Leiden, The Netherlands; 2 Angiogenesis Laboratory, Department of Medical Oncology, VU University Medical Center, Amsterdam, The Netherlands; 3 Department of Molecular Cell Biology, Leiden University Medical Center, Leiden, The Netherlands; 4 Department of Gynecology, Leiden University Medical Center, Leiden, The Netherlands; 5 Center for Gynecological Oncology Amsterdam, VU University Medical Center, Amsterdam, The Netherlands; Gustave Roussy, FRANCE

## Abstract

Galectins are proteins that bind β-galactoside sugars and provide a new type of potential biomarkers and therapeutic targets in cancer. Galectin-1, -3 and -9 have become the focus of different research groups, but their expression and function in cervical cancer is still unclear. The aim of this study was to determine the phenotype of galectin-1, -3 and -9 expressing cells and the association with clinico-pathological parameters in cervical cancer. Galectin expression was scored in tumor cells, tumor epithelium infiltrating immune cells and stromal cells in squamous cervical cancer (n = 160). Correlations with clinico-pathological parameters and survival were studied according to the REMARK recommendations. We additionally investigated whether the galectins were expressed by tumor cells, fibroblasts, macrophages and T cells. Galectin-1 and -9 were both expressed by tumor cells in 11% of samples, while 84% expressed galectin-3. Strong galectin-1 expression by tumor cells was an independent predictor for poor survival (hazard ratio: 8.02, p = 0.001) and correlated with increased tumor invasion (p = 0.032) and receiving post-operative radiotherapy (p = 0.020). Weak and positive tumor cell galectin-3 expression were correlated with increased and decreased tumor invasion, respectively (p = 0.012). Tumor cell expression of galectin-9 showed a trend toward improved survival (p = 0.087). The predominant immune cell type expressing galectin-1, -3 and -9 were CD163^+^ macrophages. Galectin-1 and -3 were expressed by a minor population of T cells. Galectin-1 was mainly expressed by fibroblasts in the tumor stroma. To conclude, while tumor cell expression of galectin-9 seemed to represent a beneficial response, galectin-1 expression might be used as a marker for a more aggressive anti-cancer treatment.

## Introduction

Cervical cancer is caused by high risk human papillomavirus (HPV) infection [1]. The mortality rate has declined by 80% in the 20^th^ century, mainly by the introduction of screening for the prevention and early detection of cervical cancer [2]. Despite this progress, cervical cancer is still the second leading cause of death by cancer in young women worldwide. Further research is thus required to select prognostic biomarkers and therapeutic targets. Potential new targets are galectins, proteins that bind β-galactoside-containing glycans via one or more carbohydrate recognition domain (CRD) [3]. In recent years it has become evident that galectins play an important role in tumor progression by regulating immune cell homeostasis [4], tumor metastasis [5], and tumor angiogenesis [6].

The most studied galectin types so far are galectin-1, -3 and -9. Galectin-1 consists of one homodimerizing CRD which homodimerizes and is expressed in most organs and by macrophages, T and B cells [4]. Galectin-1 increases cellular growth and motility and binds cells to the extracellular matrix (ECM) as well as to other cells [7]. In the tumor microenvironment, galectin-1 induces angiogenesis [4,8] and may facilitate metastasis by binding tumor cells to endothelial cells. Functioning as a weak T cell receptor ligand, apoptosis is induced in activated T cells [4,7]. Galectin-3 is a chimeric galectin containing a CRD and an N-terminal non-CRD domain. Galectin-3 is expressed by macrophages, fibroblasts, activated T cells, eosinophils, epithelial and tumor cells, inducing anti-apoptotic signaling [4]. Expression is associated with a differentiated phenotype and ECM adhesion regulation [9]. Like galectin-1, galectin-3 has also been linked to increased angiogenesis [10] and metastasis [11]. Extracellular galectin-3 can bind T cells, neutrophils and macrophages [9]. In T cells, galectin-3 expression has been shown to promote survival when expressed intracellularly, but to induce apoptosis when present extracellularly [12]. Galectin-9 contains two CRDs connected by a linker peptide of variable length. Galectin-9 can be expressed by epithelial cells as well as immune cells including T cells and neutrophils [13]. The protein acts as an eosinophil chemoattractant while intracellular expression has been reported to induce apoptosis in activated T cells, potentially via T cell immunoglobulin mucin-3 (TIM-3), leading to inhibition of T helper 1 (Th1) and Th17 cells and stimulation of regulatory T cells (Tregs) [14,15]. Galectin-9 expression has also been reported in endothelial cells but the role of this protein in angiogenesis appears to be limited [16].

The involvement of galectins in different processes of tumor progression is supported by reports that altered galectin expression has diagnostic or prognostic value in different cancer types including ovarian, prostate, breast, head and neck and non-small cell lung cancer [17–26]. In squamous cervical cancer patients who received radiation therapy, a recent study reported that expression of galectin-1 by the tumor was an independent predictor for local recurrence and poor survival [27]. Expression of galectin-1 in the stroma of cervical cancer has also been correlated with higher histopathological grade [28] and lymph node metastasis [29]. Information about galectin-3 and galectin-9 expression in cervical cancer is limited. Lee et al described an inverse association between galectin-3 [30] and tumor grade, while galectin-9 expression has been shown to be positively correlated with tumor differentiation grade in squamous cervical cancer [31].

To get better insight in the role of galectins in cervical cancer, the aim of this study was to determine whether the expression of galectin-1, -3 and -9 is associated with survival in a squamous cervical cancer cohort (n = 160). Expression of the different types of galectins by tumor cells and by tumor epithelium and stroma infiltrating cells were scored. We also investigated which cancer-associated stromal (CAS) cells (fibroblasts, macrophages and T cells) expressed these galectins.

## Materials and Methods

### Patient material

Formalin-fixed, paraffin-embedded (FFPE) squamous cervical cancer specimens obtained from all patients who underwent primary surgical treatment for cervical cancer between 1985 and 2005 with sufficient material available for analysis as described before [32], were retrieved from the archives of the Department of Pathology, Leiden University Medical Center (n = 160). None of the patients had received preoperative anticancer therapy. Disease-free and disease-specific survival time were defined as the period of time in months from the date of surgery to the date of relapse and death from cervical cancer, respectively. Patients who died from other causes were censored in survival analyses. Patient and tumor characteristics are listed in S1 Table. Patient samples were handled according to the medical ethical guidelines described in the Code of Conduct for Proper Secondary Use of Human Tissue of the Dutch Federation of Biomedical Scientific Societies. Patients receive information on the secondary use of the tissue sampled for diagnostic use and can actively object to secondary use. According to the guidelines, all human material used in this study has been anonymized. Because of the anonymization, retrospective research does not require ethical approval from the Institutional Review Board and individual consent is not required.

### Immunofluorescent stainings

Triple immunofluorescent staining was performed on 4 μm thick FFPE sections of all samples. After antigen retrieval using Tris-EDTA buffer (10 mM TRIS, 1 mM EDTA pH 9.0), polyclonal rabbit anti-galectin-1 (1:1000, ab25138, RRID: AB 2136615, lot GR127498-2, Abcam, Cambridge, UK), monoclonal rat anti-galectin-3 (1:50, clone M3/38, RRID: AB 1134237, lot B160878, BioLegend, London, UK) and polyclonal goat anti-galectin-9 (1:200, AF2045, RRID: AB 2137232, lot KNI0112011, R&D Systems, Abingdon, UK) diluted in 1% w/v bovine serum albumin (BSA) in phosphate buffered saline (PBS) were incubated at room temperature overnight. Alexa Fluor labelled donkey anti-rabbit-A647 (A31573, RRID: AB 10561706, lot 1069817), donkey anti-rat-A488 (A21208, RRID: AB 10562718, lot 1081955) and donkey anti-goat-A546 (A11056, RRID: AB 10584485, lot 997810; all polyclonal, 1:200, Invitrogen, Life Technologies, Carlsbad, USA) were incubated at room temperature for one hour. Slides were mounted using Mowiol mounting medium [33].

Double immunofluorescent stainings were performed on three samples with above median and three samples with below median stromal expression of galectin-1, -3 and -9. Rabbit anti-galectin-1 and rat anti-galectin-3 were combined with mouse IgG1 anti-CD163 (1:400, clone 10D6, RRID: AB 563510, lot 6002913, Leica Microsystems, Newcastle, UK) and mouse IgG1 anti-CD3 (1:25, clone F7.2.38, Dako, Glostrup, Denmark) followed by Alexa Fluor labelled goat-anti-rabbit-A488 (A11008, RRID: AB 10563748, lot 828814) or goat-anti-rat-A488 (A11006, RRID: AB 10561520, lot 421559) and goat-anti-mouse IgG1-A546 (A21123, RRID: AB 10562376, lot 872637) or goat-anti-mouse IgG1-A647 (A21240, RRID: AB 10565021, lot 1248996). Goat anti-galectin-9 was also combined with mouse IgG1 anti-CD163 and anti-CD3 and followed by donkey-anti-goat-A488 (A11055, RRID: AB 10564074, lot 55308A) and donkey-anti-mouse IgG-A647 (A31571, RRID: AB 10584497, lot 1069838; all polyclonal, 1:200, Invitrogen). Slides were mounted using VectaShield mounting medium containing DAPI (Vector Laboratories, Burlingame, USA). Normal cervix and vulva tissue were used as positive control. Omitting one or more of the primary antibodies or substituting them for antibodies of the same isotype class with an unknown specificity were used as negative controls.

### Microscopic analysis

Images of the galectins triple staining were digitized with a Pannoramic Midi automated slide scanner and analyzed using Pannoramic Viewer (3DHISTECH, Budapest, Hungary). A fixed exposure time was determined per slide for each of the three fluorescent labels. Brightness and contrast were adjusted for each slide based on non-tumor cells with a known staining intensity. At least three but generally four or five random images were taken at a 200x magnification, sampling a total tumor area of 1.1 to 1.9 mm^2^ of each slide, comprising both tumor epithelium and stroma. Expression by tumor cells was scored without knowledge of clinico-pathological parameters as described by Ruiter et al [34]. Intensity was scored as absent (0), weak (1), moderate (2) or intense (3). Moderate and intense staining intensity were considered strong. The percentage of positive cells was scored as 0% (0), 1–10% (1), 11–25% (2), 26–50% (3), 51–75% (4) and 76–100% (5). A staining with a combined score above 2 was considered positive.

Since not only tumor cells, but also CAS cells within the tumor epithelium and stroma expressed galectin-1, -3 and -9, total expression within the tumor epithelium and stroma was analyzed by the in-house image analysis software program Stacks version 2.1 (Dr. J. Vrolijk, Ing. W. Sloos, Department of Molecular Cell Biology, Leiden University Medical Center, Leiden, the Netherlands; unpublished software). Positive staining thresholds were determined by comparing the threshold image with the original staining in Pannoramic Viewer. Stromal areas were marked and scored separately from the epithelium. Blood vessels, autofluorescence and necrotic areas were manually removed from the analysis. The total area and the number of single, double and triple positive pixels were obtained. Ten random samples were scored manually for the percentage of single, double and triple positive cells in the tumor epithelium and stroma, which corresponded with the analyses using Stacks.

The distribution of single and double positive cells in the double stainings was scored in categories: <5%, 5–50%, >50% or practically all cells positive in at least four areas per slide using an LSM700 confocal laser scanning microscope equipped with an LCI Plan-Neofluar 25x/0.8 Imm Korr DIC M27 objective (Zeiss, Göttingen, Germany). Images were acquired using a C-Apochromat 40x/1.20 W Korr objective (Zeiss).

### Statistical analysis

Statistical analyses were performed using SPSS version 20.0 (IBM Corp., Armonk, USA). Correlations (R) between the galectin expression scores by both the Ruiter and Stacks analyses were tested using Spearman’s rank correlation rho test. Based on scatterplots, correlations with R>0.45 were considered positive and correlations with R>0.67 strong. Correlations between galectin expression and clinico-pathological variables were studied using Pearson Chi-Square and Fisher’s Exact Test. Survival correlations were tested using the Kaplan-Meier and Cox proportional hazards models, where p-values below 0.05 were considered statistically significant.

## Results

### Expression of galectin types 1, 3 and 9 in squamous cervical cancer

**Fig 1:**
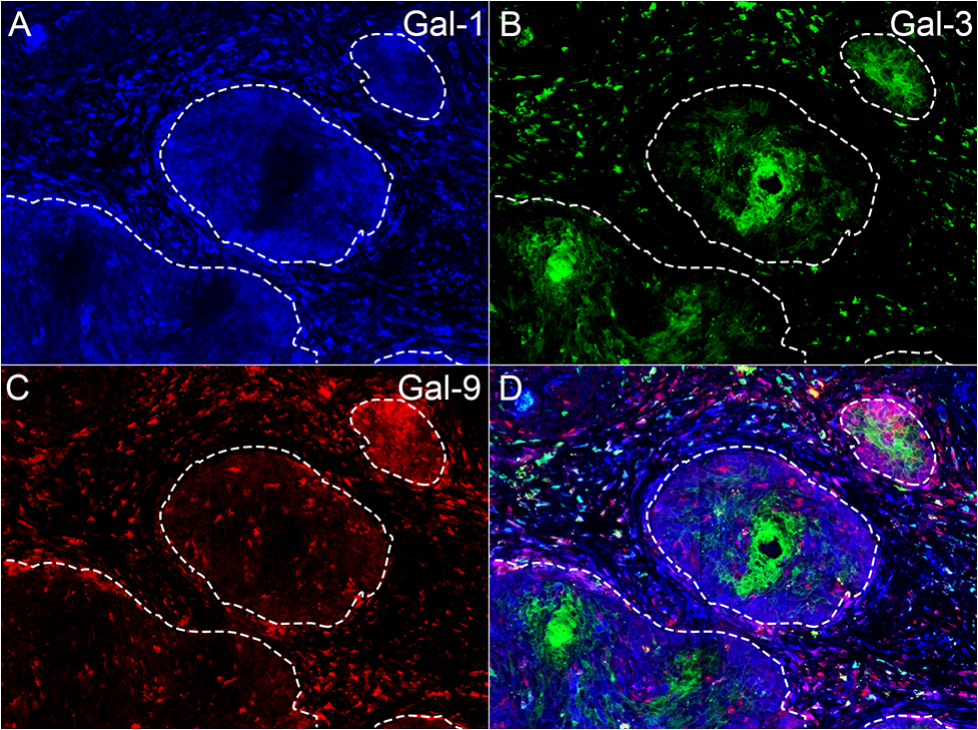
Immunofluorescent staining of galectin-1, -3 and -9. Representative triple staining image in an FFPE squamous cervical cancer sample containing tumor epithelial cells expressing galectin-1 (A, blue), galectin-3 (B, green) and galectin-9 (C, red) at a 200x magnification. Tumor epithelial fields are marked by dashed lines. Typically, galectin-1 was expressed in a high number of stromal cells and could be weakly expressed by tumor epithelial cells. Galectin-3 was expressed in stromal cells and often either in epithelial field centers (as shown here) or in small groups of epithelial cells at the invasive border. Galectin-9 could also be expressed by both stromal and epithelial cells. When galectin-1 or -9 were expressed by epithelial cells, this was typically observed at the borders of tumor fields, while galectin-3 was expressed in the center, as demonstrated in this figure. Tumor infiltrating CAS cells expressing galectin-1, -3 and -9 were also frequently observed within the tumor epithelial fields.

**Fig 2:**
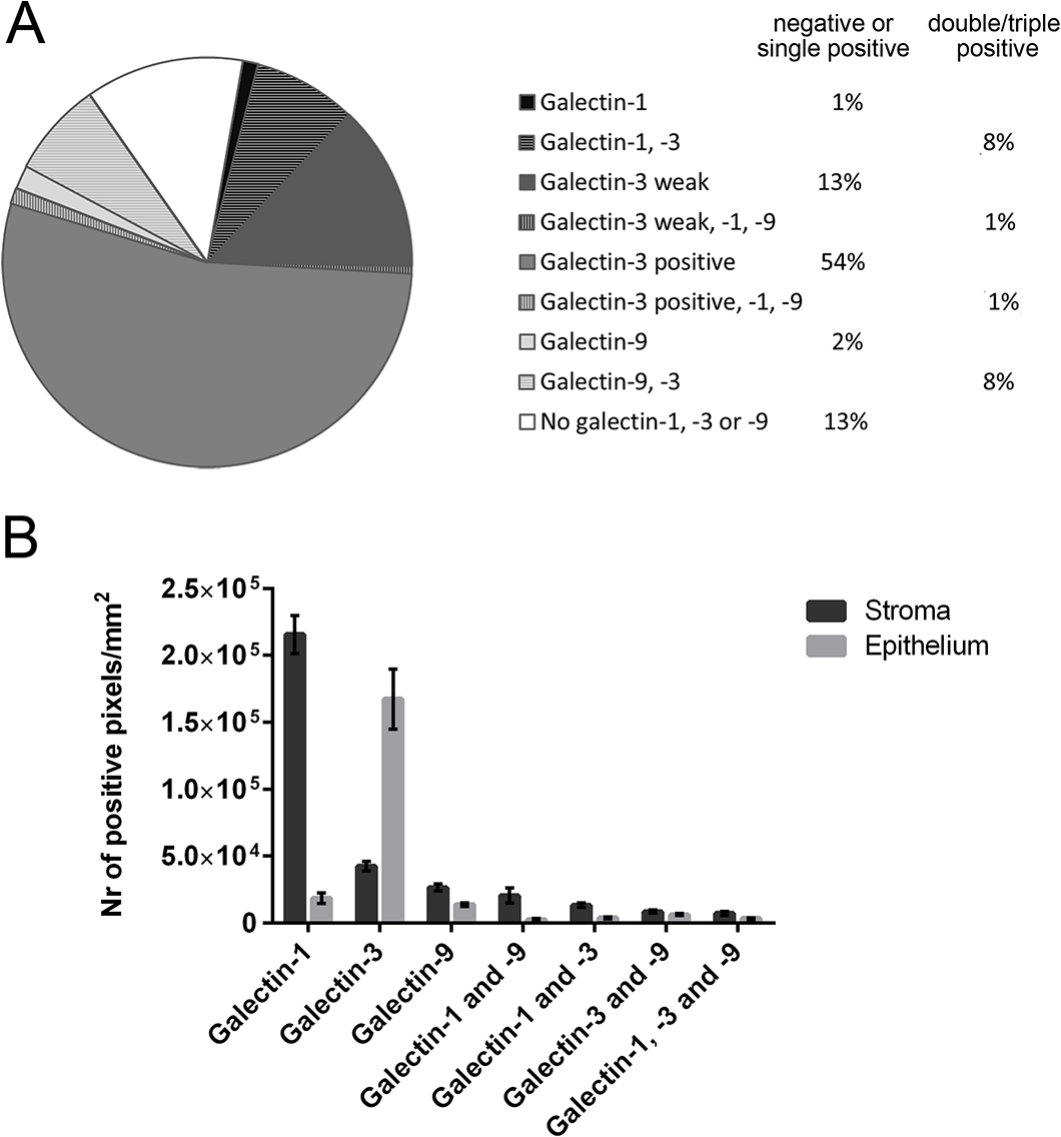
Distribution of galectin expression. The fraction of samples in which tumor cells expressed galectin-1, -3 or -9 or a combination is shown in A. The fraction of samples positive for one or none of the galectin types studied is shown in the first percentages column, while fractions of samples expressing two or more galectin types are shown in the second column. Total galectin-1, -3 and -9 single, double and triple positive pixels in the tumor stroma and tumor epithelium is represented in B. Mean and standard error of the mean are displayed.

Galectin-1, -3 and -9 were expressed in the cytoplasm of different cell types (Fig 1). The expression of the different galectin types by tumor epithelial cells was quantified by the Ruiter score. Tumor epithelial cells in most samples did not express galectin-1 (89% of samples) or galectin-9 (89% of samples), see Fig 2A. To assess all galectin expressing cells, galectin expression was assessed in both the tumor epithelial and stromal compartment using the in-house image analysis software program Stacks. Galectin-1 was generally present in a high number of stromal cells (Fig 2B). Variable expression of galectin-3 was observed both in tumor cells and stromal cells. Expression of galectin-3 was found in 84% of the samples. Strong tumor cell galectin-3 expression was often present either at the invasive border or center of the tumor fields. In tumor cells, galectin-3 expression often appeared mutually exclusive with expression of galectin-1 or -9, since its expression was reduced when expression of another type of galectin was present. If this was the case, galectin-3 was mainly expressed in the center of the tumor fields, while galectin-1 and -9 were expressed at the invasive border. Cells expressing galectin-9 were generally not abundant in either the tumor epithelium or the stromal cells. When galectin-9 was expressed by tumor epithelial cells, it was primarily located at the invasive borders.

### Correlation between galectin-1, -3 and -9 expression

All galectin expressing single, double and triple positive cells in the tumor stroma were significantly correlated with the same cells in the tumor epithelium (0.57<R>0.72, p<0.0001, S2 Table), except for galectin-3 single positive cells, the only galectin type studied that was expressed by tumor cells in the majority of the samples. This was also the only galectin type for which epithelial single positive scores were strongly correlated with the Ruiter score of the tumor cell expression (R = 0.743, p<0.0001, S1 Fig). Single positive galectin-1, -3 and -9 were not significantly correlated with each other. Strong correlations were found between galectin-9 single positive cells and galectin-3/9 double positive cells both in the epithelial and in the stromal tumor compartment (R = 0.706, p<0.0001; R = 0.680, p<0.0001, respectively). Galectin-3 single positive cells were strongly correlated with galectin-1/3 double positive cells within the tumor stroma (R = 0.693, p<0.0001). Triple positive cells were strongly correlated with galectin-1/9 double positive cells within the tumor epithelium (R = 0.682, p<0.0001). Triple positive cells in the stroma were correlated with galectin-3/9 double positive cells (R = 0.704, p<0.0001).

### Phenotype of galectin expressing cells

**Fig 3:**
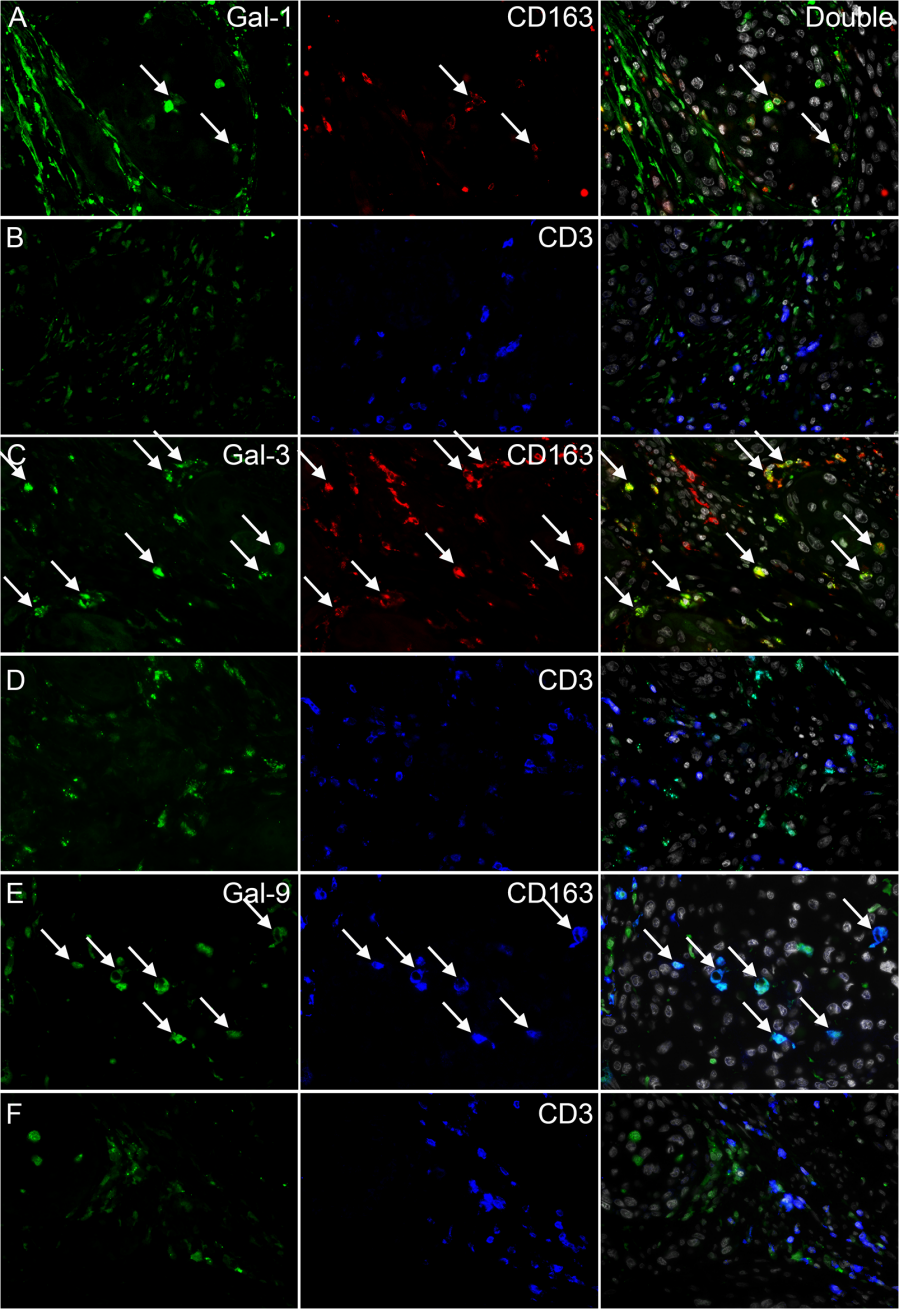
Immunofluorescent stainings of galectin-1, -3, -9, CD163 and CD3. Representative images from double stainings of galectin-1 and CD163 (A) or CD3 (B), galectin-3 and CD163 (C) or CD3 (D) and galectin-9 and CD163 (E) or CD3 (F) are shown. Images containing both stainings and DAPI are shown in the right column. Arrows indicate examples of double positive cells.

To study whether macrophages and T cells expressed galectin-1, -3 and -9, we performed double stainings with type 2 macrophage marker CD163 or T cell marker CD3 (Fig 3). Galectin-1 expressing cells were not abundant in the tumor epithelium, but when present the majority was CD163^+^. A minority of the galectin-1 expressing cells was CD3^+^. Most stromal galectin-1 expressing cells were not expressing CD163 or CD3 and were termed fibroblasts based on cellular morphology.

Almost all non-epithelial cells expressing galectin-3 were CD163^+^ macrophages. The reverse was not true, as we also observed a large number of CD163^+^ cells that did not express galectin-3. A minor population of galectin-3 expressing cells was CD3^+^.

The majority of galectin-9 expressing cells was CD163^+^ and almost all CD163^+^ macrophages were galectin-9^+^. We did not observe CD3^+^ galectin-9 expressing cells.

Although correlations between the galectins and angiogenesis have been described, we did not observe a clear vessel staining or a similar morphology compared with a staining for CD105 (S2 Fig). Since we also did not find a correlation with the number of CD105^+^ vessels (data not shown), galectin-1, -3 and -9 were not abundantly expressed by endothelial cells in this study cohort.

### Correlation between galectin expression and clinico-pathological parameters

**Fig 4:**
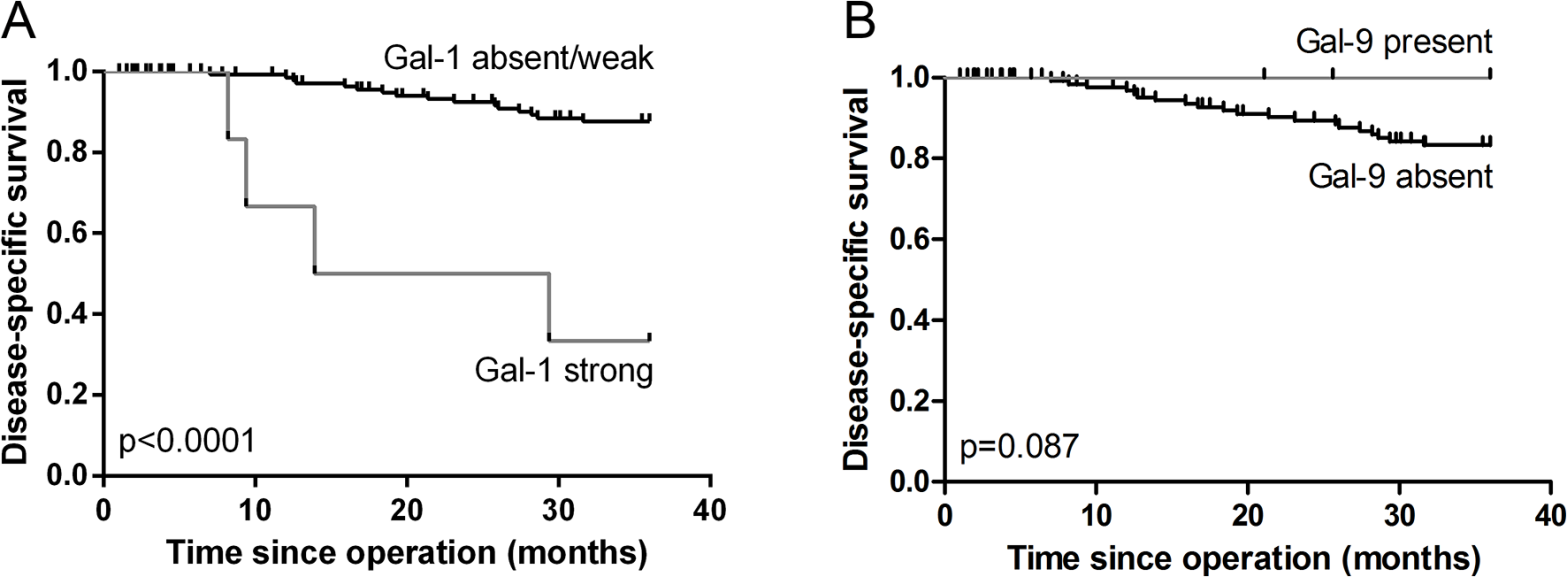
Survival analyses for galectin-1, -3 and -9 expression. Kaplan-Meier survival curve for strong compared with absent to weak expression of galectin-1 by tumor cells is shown in A. A survival analysis comparing patients with present versus absent tumor expression of galectin-9 is shown in B.

Squamous cervical cancer patients with TNM stage I (n = 110), II (n = 44), III (n = 4) and IV (n = 2) were included in this study. The median age was 45 years (range 22–87) and twenty patients died from the disease within three years. Strong expression of galectin-1 by tumor cells was significantly correlated with poor disease-free (p = 0.0004) and disease-specific survival (p<0.0001, Fig 4A). The hazard ratio for disease-specific survival in case of strong galectin-1 tumor expression was 8.9 (95% CI: 2.961–26.717, Table 1). Tumor cell galectin-1 expression was also correlated with increased tumor invasion depth (p = 0.032, Table 2). Probably related to this, patients with tumors expressing galectin-1 more frequently received post-operative radiotherapy (p = 0.020). Radiotherapy was administered to 16 out of 18 patients with any expression of galectin-1 and to all six patients with strong expression of galectin-1.

**Table 1:** Cox regression analyses.

	Univariate		Multivariate	
Variable	Hazard ratio (95% CI)	p-value	Hazard ratio (95% CI)	p-value
FIGO stage	1.397 (0.560–3.482)	0.473	1.453 (0.567–3.723)	0.436
Lymph nodes	2.117 (0.877–5.110)	0.096	1.769 (0.688–4.544)	0.236
Vaso-invasion	1.206 (0.481–3.023)	0.689	0.739 (0.266–2.055)	0.562
Galectin-1 tumor intensity	8.895 (2.961–26.717)	**<0.0001**	8.017 (2.460–26.127)	**0.001**

fn id="t001fn001">Univariate and multivariate Cox regression analyses for FIGO stage, presence of lymph node metastasis and vaso-invasion and moderate/strong versus absent/weak tumor galectin-1 expression on disease-specific survival are shown. Bold numbers indicate a correlation with p<0.05.

**Table 2:** Tumor galectin expression and clinico-pathological parameters.

		Tumor size		p value	Invasion depth		p value	Vaso-invasion		p value	Lymph node metastasis		p value	Postoperative Radiotherapy		p value	HPV status		p value
		<40 mm	≥40 mm		<15 mm	≥15 mm		no	yes		no	yes		no	yes		**16/18**	**other**	
**Galectin-1**	absent	55	68	1.000	77	59	**0.032**	60	80	0.793	95	46	0.793	55	87	**0.020**	113	29	0.230
	present	7	9		4	11		6	10		13	5		2	16		12	6	
**Galectin-3**	absent	8	14		12	12		13	12	0.497	14	11		9	16		16	9	
	weak	16	12	0.284	7	18	**0.012**	9	16		17	11	0.184	7	21	0.424	17	11	**0.003**
	positive	38	51		62	40		44	62		77	29		41	66		92	15	
**Galectin-9**	absent	53	71	0.273	71	63	0.798	62	77	0.122	95	46	0.793	53	89	0.297	107	35	**0.014**
	present	9	6		10	7		4	13		13	5		4	14		18	0	

Correlations between tumor galectin-1, -3 and-9 expression and critical clinico-pathological parameters. Bold numbers indicate a correlation with p<0.05.

Tumor expression of galectin-9 showed a trend toward improved survival (p = 0.087, Fig 4B). Interestingly, while weak tumor galectin-3 expression was correlated with increased invasion depth, positive expression was correlated with decreased tumor invasion (p = 0.012, Table 2). Both galectin-3 and galectin-9 expression were significantly correlated with the presence of HPV type 16 or 18. Tumor size and the presence of vaso-invasion or lymph node metastasis were not significantly different between samples with or without expression of galectin-1, -3 or -9 either in the tumor epithelium or stroma.

Multivariate analysis of disease-specific survival was performed for tumor galectin-1 intensity by correcting for FIGO stage, lymph node metastasis and vaso-invasion (Table 1). FIGO stage was used because this covered tumor size and infiltration depth and was known for a larger number of patients than the separate variables. Lymph node metastasis and vaso-invasion are two other parameters known to influence cervical cancer patient prognosis. Galectin-1 tumor intensity independently correlated with poor survival, with a hazard ratio of 8.0. When FIGO stage and lymph node metastasis status were replaced by TNM stage, the correlation remained statistically significant (hazard ratio: 6.84, 95% CI: 2.013–23.256, p = 0.002). Tumor galectin-1 intensity was also an independent predictor of poor disease-free survival (hazard ratio: 3.41, 95% CI: 1.296–8.972, p = 0.013). Data were available for 155 patients, including 41 relapses and 30 deaths.

## Discussion

The present study investigated the correlations between expression of galectin-1, -3 and -9 and survival in a squamous cervical cancer cohort (n = 160) following the REMARK recommendations for prognostic tumor marker studies [35]. Galectin expression by tumor cells and tumor epithelium and stroma infiltrating cells were scored using two complementary scoring systems. We also investigated which cancer-associated stromal (CAS) cells (fibroblasts, macrophages and T cells) expressed these galectins.

Since tumor cell expression of galectin-1 and -9 was generally low or absent, galectin-1 and -9 in tumor epithelial fields were predominantly expressed by tumor epithelium infiltrating immune cells. This also explains the absence of a strong correlation between total expression and the Ruiter score for galectin-1 and -9, while epithelial galectin-3 single positivity was strongly correlated with the Ruiter score for galectin-3. The correlations also indicate that the two scoring methods used were not only complementary but also validated each other.

Galectin-1, -3 and -9 were expected to be expressed by macrophages, T cells, epithelial cells and fibroblasts [4,13]. Most galectin-1 expressing cells infiltrating in the tumor epithelium were macrophages, predominantly CD163^+^ type 2 macrophages. A minor population consisted of galectin-1 expressing T cells, although more galectin-1 negative T cells were in close contact with the galectin-1 positive cells. This corresponds with the proposed function of galectin-1 in inducing T cell apoptosis resulting in immunosuppression [4,7]. The amount of galectin-3 expression by CAS cells was highly variable between patients, but this galectin type was practically always expressed by CD163^+^ type 2 macrophages. This corresponds with an *in vitro* study showing that galectin-3 is more frequently expressed in type 2 than type 1 macrophages and also to a higher extent than galectin-1 [36]. Galectin-3 was thus predominantly expressed by type 2 macrophages, suggesting that tumor infiltrating cells expressing galectin-3 might be associated with a tumor promoting microenvironment. This corresponds with its reported functions in promoting cell survival [4] and angiogenesis [10]. Since galectin-1 was practically always expressed by tumor infiltrating immune cells and galectin-3 among non-tumor cells by CD163^+^ macrophages, the galectin-1/3 double positive cells are expected to be tumor epithelium infiltrating CD163^+^ macrophages. Contact between galectin-3 expressing cells and T cells was more evident than for galectin-1, which again might induce T cell apoptosis [12]. A study by Hirashima et al. has described that galectin-9 is expressed by T cells and neutrophils [13]. In contrast, we have shown that this galectin is predominantly expressed by macrophages in cervical cancer. Additionally, almost all CD163^+^ macrophages were galectin-9^+^ and we did not observe galectin-9 expressing T cells. The function of galectin-9 in tumor associated macrophages requires further investigation.

Galectin-1 has been described to be infrequently expressed by tumor cells [19,27,28], which was confirmed by the current study. Patients with strong tumor cell galectin-1 expression had increased tumor invasion and received post-operative radiotherapy treatment more frequently. Tumor galectin-1 expression was an independent predictor of poor disease-specific survival. This suggests that galectin-1 expression is associated with more aggressive tumor growth. This corresponds with a study by Huang et al., who reported on a correlation between tumor expression of galectin-1 and poor survival in cervical cancer patients treated with curative-intent radiation therapy [27]. Indeed, increased galectin-1 expression is generally associated with poor prognosis in different tumor types [17,18,26,27]. Galectin-1 may increase tumor progression and invasiveness [37]. The present study, performed in a consecutive cohort of squamous cervical cancer patients, shows that tumor galectin-1 is a marker for poor survival.

Inconsistent results have been described for galectin-3, which has been associated with good prognosis [17,38] as well as poor prognosis in different tumor types [24,39]. In cervical neoplasia, galectin-3 has been shown to be inversely correlated with progression [30]. Our results suggest that strong galectin-3 expression is correlated with less tumor growth, while weak galectin-3 may be correlated with increased tumor growth. Tumor galectin-3 expression may be necessary for a differentiated phenotype and ECM adhesion [9]. The function of galectin-3 in promoting cell survival [4] and potentially inducing chemo-resistance [40] and T cell apoptosis [12] might explain the correlation between expression and poor prognosis. Because the functions and correlations with clinico-pathological parameters and survival of galectin-3 are so diverse, it will be challenging to use this galectin type as a prognostic or diagnostic marker in cervical cancer.

The expression of galectin-9 by tumor cells has generally been correlated with good prognostic markers in different tumor types [17,22,23,26,41]. The role of the different types of galectins seems to be tumor type dependent though [41], and galectin-9 has been correlated with poor survival in clear-cell renal cell carcinoma [42]. In cervical cancer, a correlation with high differentiation grade has been described for galectin-9 expression [31], in agreement with the trend toward improved prognosis described in our study. Tumor galectin-9 expression might be related to HPV infection, as different viruses including human cytomegalovirus, HIV and influenza virus have been shown to induce galectin-9 expression [43–45]. We found that galectin-3 and galectin-9 expression were both significantly correlated with HPV16 or HPV18 expression, supporting this hypothesis.

To conclude, tumor galectin-1 expression might be used as a marker for a more aggressive anti-cancer treatment. Galectin-1 and -3 were most likely co-expressed by tumor epithelium infiltrating type 2 macrophages. Tumor galectin-3 might have dual functions, as weak expression was correlated with increased tumor invasion, while positive expression was correlated with decreased invasion. Tumor expression of galectin-9 showed a trend toward improved survival, which might be related to virus induced expression. As blocking galectin-1 expression has already been shown to inhibit tumor angiogenesis [8] and induce T cell dependent tumor rejection in mice [46], our data support further studies of the therapeutic potential of targeting galectin-1 in positive tumors.

## Supporting Information

S1 Fig Epithelial Stacks versus Ruiter scores. (DOCX)

S2 Fig Galectin and vessel staining. (DOCX)

S1 Table Patient and tumor characteristics. (DOCX)

S2 Table Correlations between galectin expressions. (DOCX)
