# Supplementary material for: Galectin-1, -3 and -9 Expression and Clinical Significance in Squamous Cervical Cancer
Source: PLoS One. 2015 Jun 12;10(6):e0129119. doi: 10.1371/journal.pone.0129119 (PMC4467041; doi:10.1371/journal.pone.0129119)
Supplement: S1 Fig — (DOCX) [file pone.0129119.s001.docx]

**
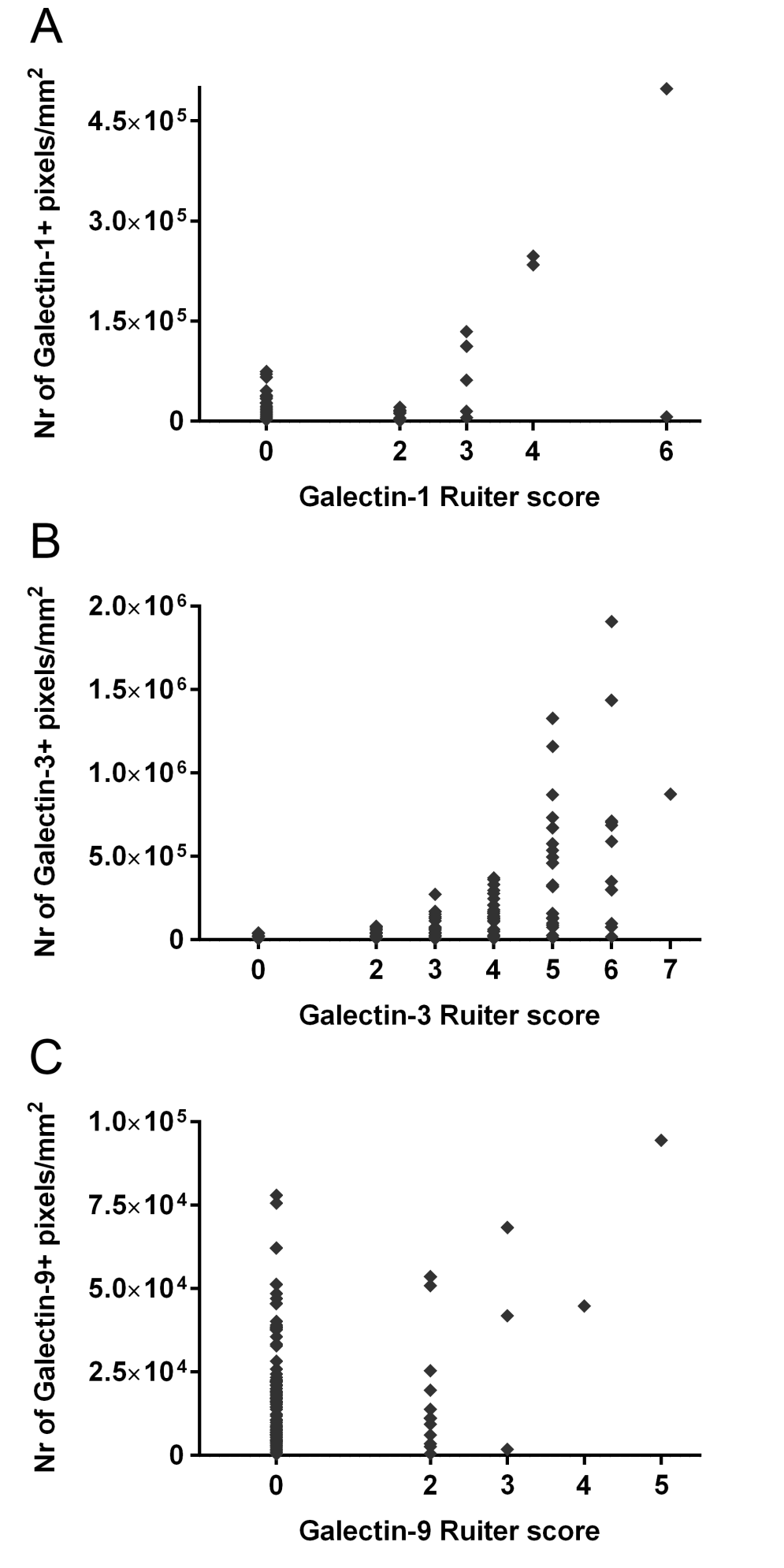
**

The total number of pixels scored in the tumor epithelium using Stacks was compared with the combined percentage and intensity scoring of galectin expressing tumor cells using the Ruiter score. Total galectin-1 single positivity was weakly correlated with tumor cell expression (R=0.247, p=0.002). Total galectin-3 single positivity was strongly correlated with tumor cell expression (R=0.743, p<0.0001). Total galectin-9 single positivity was not correlated with tumor cell expression (R=0.143, p=0.075).
