# Supplementary material for: Galectin-1, -3 and -9 Expression and Clinical Significance in Squamous Cervical Cancer
Source: PLoS One. 2015 Jun 12;10(6):e0129119. doi: 10.1371/journal.pone.0129119 (PMC4467041; doi:10.1371/journal.pone.0129119)
Supplement: S2 Fig — (DOCX) [file pone.0129119.s002.docx]

**
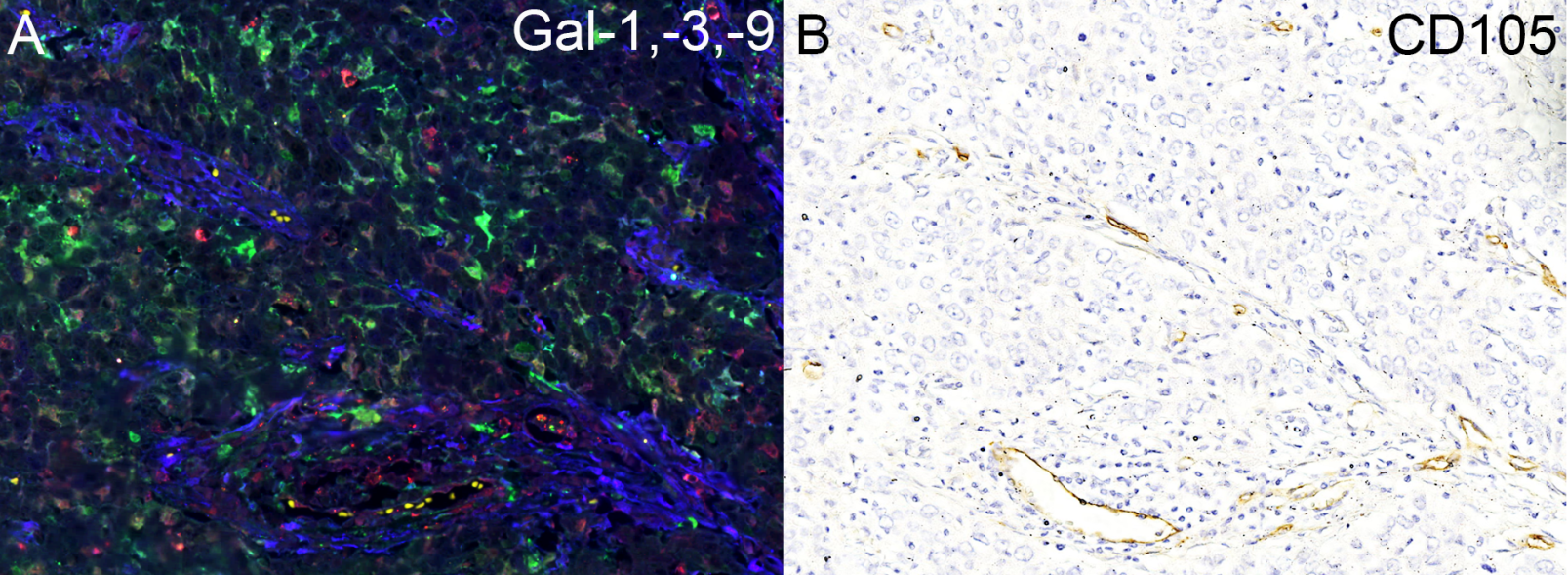
**

The staining for galectin-1, -3 and -9 (A) is compared with a staining on the same tissue sample for activated endothelium marker endoglin (CD105, B). While many galectin-1 expressing cells (blue) had a spindle-shaped morphology, we did not observe a similar staining pattern for endoglin. Most other galectin expressing cells had a macrophage morphology and did not express the clear vascular shape that endoglin expressed either. Although some vessels may express galectins, vessel expression of galectin-1, -3 and -9 expression did not seem abundant in squamous cervical cancer.
