# Supplementary material for: Galectin-1, -3 and -9 Expression and Clinical Significance in Squamous Cervical Cancer
Source: PLoS One. 2015 Jun 12;10(6):e0129119. doi: 10.1371/journal.pone.0129119 (PMC4467041; doi:10.1371/journal.pone.0129119)
Supplement: S1 Table — (DOCX) [file pone.0129119.s003.docx]

| **Clinicopathological parameter** | **Category** | **N = 160 (%)** |
| --- | --- | --- |
| **Age** | Median | 45 |
|  | Range | 22-87 |
| **FIGO stage***† | IB | 122 (76) |
|  | IIA | 36 (23) |
|  | IIB | 1 (1) |
| **TNM stage** | IB1 | 46 (29) |
|  | IB | 19 (12) |
|  | IB2 | 45 (28) |
|  | IIA | 30 (19) |
|  | IIB | 14 (9) |
|  | IIIA | 1 (1) |
|  | IIIB | 3 (2) |
|  | IV | 2 (1) |
| **Lymph nodes**† | negative | 108 (68) |
|  | positive | 51 (32) |
| **Tumor size (mm)**† | <40 | 62 (39) |
|  | ≥40 | 77 (48) |
| **Vaso-invasion**† | Absent | 66 (41) |
|  | Present | 90 (56) |
| **Infiltration depth (mm)**† | <15 | 81 (51) |
|  | ≥15 | 70 (44) |
| **HPV type** | 16 | 97 (61) |
|  | 18 | 28 (18) |
|  | other | 35 (22) |

*FIGO, International Federation of Gynecologists and Obstetricians

†For some variables, data were not available for all patients.
