# Supplementary material for: Galectin-1, -3 and -9 Expression and Clinical Significance in Squamous Cervical Cancer
Source: PLoS One. 2015 Jun 12;10(6):e0129119. doi: 10.1371/journal.pone.0129119 (PMC4467041; doi:10.1371/journal.pone.0129119)
Supplement: S2 Table — (DOCX) [file pone.0129119.s004.docx]

|  |  | Stroma | | | | | | | Epithelium | | | | | |
| --- | --- | --- | --- | --- | --- | --- | --- | --- | --- | --- | --- | --- | --- | --- |
|  |  | **Galectin-1** | **Galectin-3** | **Galectin-9** | **Galectin-1/9** | **Galectin-1/3** | **Galectin-3/9** | **Galectin-1/3/9** | **Galectin-1** | **Galectin-3** | **Galectin-9** | **Galectin-1/9** | **Galectin-1/3** | **Galectin-3/9** |
| Stroma | **Galectin-3** | 0.017 (0.831) |  |  |  |  |  |  |  |  |  |  |  |  |
|  | **Galectin-9** | 0.099 (0.218) | -0.056 (0.485) |  |  |  |  |  |  |  |  |  |  |  |
|  | **Galectin-1/9** | 0.464 (<0.0001) | -0.075 (0.354) | 0.600 (<0.0001) |  |  |  |  |  |  |  |  |  |  |
|  | **Galectin-1/3** | 0.441 (<0.0001) | 0.693 (<0.0001) | -0.089 (0.274) | 0.098 (0.225) |  |  |  |  |  |  |  |  |  |
|  | **Galectin-3/9** | 0.1 (0.218) | 0.447 (<0.0001) | 0.706 (<0.0001) | 0.331 (<0.0001) | 0.334 (<0.0001) |  |  |  |  |  |  |  |  |
|  | **Galectin-1/3/9** | 0.426 (<0.0001) | 0.432 (<0.0001) | 0.447 (<0.0001) | 0.551 (<0.0001) | 0.628 (<0.0001) | 0.704 (<0.0001) |  |  |  |  |  |  |  |
| Epithelium | **Galectin-1** | 0.572 (<0.0001) | 0.062 (0.442) | 0.089 (0.268) | 0.336 (<0.0001) | 0.428 (<0.0001) | 0.06 (0.458) | 0.301 (0.0002) |  |  |  |  |  |  |
|  | **Galectin-3** | 0.254 (0.001) | -0.007 (0.927) | -0.011 (0.888) | 0.026 (0.747) | 0.099 (0.222) | 0 (0.996) | 0.092 (0.258) | 0.145 (0.071) |  |  |  |  |  |
|  | **Galectin-9** | 0.161 (0.045) | -0.05 (0.534) | 0.716 (<0.0001) | 0.414 (<0.0001) | 0.028 (0.734) | 0.545 (<0.0001) | 0.378 (<0.0001) | 0.168 (0.036) | -0.002 (0.980) |  |  |  |  |
|  | **Galectin-1/9** | 0.422 (<0.0001) | -0.027 (0.745) | 0.457 (<0.0001) | 0.671 (<0.0001) | 0.194 (0.016) | 0.283 (0.0004) | 0.505 (<0.0001) | 0.547 (<0.0001) | -0.001 (0.99) | 0.604 (<0.0001) |  |  |  |
|  | **Galectin-1/3** | 0.372 (<0.0001) | 0.384 (<0.0001) | -0.02 (0.806) | 0.126 (0.121) | 0.665 (<0.0001) | 0.171 (0.035) | 0.460 (<0.0001) | 0.621 (<0.0001) | 0.301 (0.0002) | 0.078 (0.339) | 0.351 (<0.0001) |  |  |
|  | **Galectin-3/9** | 0.146 (0.073) | 0.167 (0.04) | 0.535 (<0.0001) | 0.201 (0.013) | 0.141 (0.083) | 0.633 (<0.0001) | 0.459 (<0.0001) | 0.132 (0.103) | 0.294 (0.0003) | 0.680 (<0.0001) | 0.376 (<0.0001) | 0.279 (0.0005) |  |
|  | **Galectin-1/3/9** | 0.417 (<0.0001) | 0.191 (0.018) | 0.358 (<0.0001) | 0.386 (<0.0001) | 0.429 (<0.0001) | 0.478 (<0.0001) | 0.709 (<0.0001) | 0.403 (<0.0001) | 0.164 (0.043) | 0.512 (<0.0001) | 0.682 (<0.0001) | 0.620 (<0.0001) | 0.653 (<0.0001) |

The Spearman correlations (R) between the expression of total galectin-1, -3 and -9 single, double and triple expression in the stromal and epithelial tumor compartment obtained using Stacks. P values are shown between brackets.
